# Pancreatic Mass Requiring a Modified Endoscopic Ultrasound-Directed Transgastric Endoscopic Retrograde Cholangiopancreatography (EDGE) Procedure for Endoscopic Ultrasound (EUS) Evaluation

**DOI:** 10.7759/cureus.76875

**Published:** 2025-01-03

**Authors:** Daniel Cain, Sidart Pradeep, Samar Taqvi, Stephen Baker, Melvin Simien

**Affiliations:** 1 Internal Medicine, Baylor Scott & White All Saints Medical Center, Fort Worth, USA; 2 Pathology, Baylor Scott & White All Saints Medical Center, Fort Worth, USA; 3 Gastroenterology and Hepatology, Baylor Scott & White All Saints Medical Center, Fort Worth, USA

**Keywords:** advanced endoscopy, edge, endoscopic ultrasound, endoscopic ultrasound directed transgastric ercp, ercp, fine needle aspiration (fna), gastroenterology, pancreatic cancer

## Abstract

Pancreatic cancer is established as being a leading cause of cancer-related mortality within the U.S. annually. Endoscopic ultrasound (EUS) with fine-needle aspiration (FNA) has been shown to be more sensitive and specific when compared to computed tomography (CT) imaging for the evaluation of pancreatic masses. Patients with altered anatomy after a Roux-en-Y gastric bypass (RYGB) procedure necessitated the development of additional techniques for biopsy and evaluation of gastrointestinal structures, such as the endoscopic ultrasound-directed transgastric endoscopic retrograde cholangiopancreatography (EDGE) procedure. The ERCP procedure was enhanced with the internal EDGE, utilizing the lumen-apposing covered metal stent (LAMS) to create a gastrogastric fistula for instrumentation to pass through in order to access the excluded gastric region without any transcutaneous hardware. The case detailed here required the utilization of a modified EDGE procedure to allow for EUS-FNA of a pancreatic mass in a patient with RYBG anatomy.

This is a presentation of a modified internal EDGE procedure in a 70-year-old male with a past medical history of RYGB and colon cancer and presented with a large pancreatic mass that was unable to be sampled by a transcutaneous CT-guided biopsy or EUS alone due to his altered anatomy. Due to the RYGB anatomy of the patient, a modified internal EDGE procedure was conducted in two stages with a LAMS, followed by a transgastric EUS-FNA of the pancreatic mass after access to the isolated gastric limb was achieved. A definitive diagnosis of pancreatic adenocarcinoma was then able to be made based on this biopsy.

The benefits of using EUS for pancreatic cancer evaluation are well-established in the literature with a sensitivity of 85-92% and specificity of 96-98% when evaluating pancreatic cancers and using FNA. The pairing of this imaging modality with biopsy capability is what ultimately allowed for a viable sample to be obtained in this case.

Due to the limited available literature documenting the utility of an EDGE procedure in obtaining viable samples for oncologic evaluation, this case is a valuable addition as it demonstrates the utility of EDGE with EUS-FNA for difficult biopsy sites related to post-Roux-en-Y anatomy where more conventional measures failed. The authors believe education on the utility of EDGE procedures should be promoted and offered when appropriate and available.

## Introduction

Pancreatic cancer is the third most common cause of cancer-related mortality in the U.S. with a mortality rate of 11.1 per 100,000 people, accounting for 7% of all cancer-related deaths in the U.S. [1-3]. The annual incidence is 13.3 per 100,000 people and is slightly more common in men than women [2,3]. There are also numerous genetic syndromes associated with pancreatic cancer (e.g., hereditary breast and ovarian cancer syndrome (HBOC), hereditary nonpolyposis colorectal cancer syndrome (HNPCC), familial adenomatous polyposis (FAP), multiple endocrine neoplasia type 1 (MEN1)) that need to be considered when evaluating pancreatic cancer [4,5]. Modifiable risk factors include smoking and obesity. Comorbid conditions that have been identified as risk factors include diabetes mellitus, pancreatitis, and a history of allergies [6].

Confirmation of pancreatic mass characteristics requires biopsy and pathological evaluation for differentiation. In patients with normal gastrointestinal anatomy, an esophagogastroduodenoscopy (EGD) with endoscopic ultrasound (EUS) for visualization of the pancreas allows for biopsies to be taken during the same procedure with fine-needle aspiration (FNA). In the meta-analysis by Kitano et al., EUS was shown to be more sensitive and specific when compared to CT imaging for the evaluation of pancreatic masses, especially small pancreatic masses under 2 cm [7].

For patients who have altered anatomy after a Roux-en-Y gastric bypass (RYGB), additional endoscopic approaches have been developed for upper GI evaluation and procedures. In the last decade, the endoscopic ultrasound-directed transgastric endoscopic retrograde cholangiopancreatography (EDGE) procedure has been developed as a method to access the excluded gastric regions not easily accessible after an RYGB. This was initially done as a two-stage procedure with percutaneous endoscopic gastrostomy (PEG) placement followed by ERCP through the matured PEG site a few days later [8]. The EDGE procedure was then improved upon with the internal EDGE, utilizing the lumen-apposing covered metal stent (LAMS) to create a gastrogastric fistula for instrumentation to pass through in order to access the RYGB afferent limb without any transcutaneous hardware [9]. This is done in either a single procedure or as a staged procedure once the LAMS has been in place connecting the afferent and efferent limbs of the RYGB anatomy. The new passage created by the LAMS allows for the endoscope to reach the ampulla for ERCP via the new shorter pathway.

To the best of our knowledge, the case presented here represents one of the first published instances of using the internal EDGE procedure to perform an EUS-FNA of a pancreatic mass in a patient who previously had an RYGB. It demonstrates the utility of this approach for sampling otherwise inaccessible pancreatic masses for further evaluation and treatment planning.

This article was previously presented as a meeting abstract at the 2024 American College of Gastroenterology Annual Conference on October 28, 2024.

## Case presentation

The patient at the center of this case is a 70-year-old male with a significant past medical history of colon cancer and right hemicolectomy over 15 years ago, RYGB five years ago, reported non-alcoholic steatohepatitis, hypertension, and type 2 diabetes mellitus. His social history was significant for 12 pack-year smoking history with cessation as of 12 years ago, and four to five servings of vodka a night until two months prior. His family history was positive for pancreatic cancer in his mother and father. He presented to our facility after being referred by his gastroenterologist for concern of recurrent colon cancer on imaging, although a normal colonoscopy had been reported three years prior. He had been experiencing worsening intermittent epigastric pain with radiation to the suprapubic region for roughly six weeks at the time of presentation. He also endorsed nausea without vomiting. A physical exam on admission was significant for an ill-appearing individual with epigastric and right upper quadrant tenderness to palpation.

A CT of the abdomen and pelvis with contrast was obtained and showed a large irregular mass medial to the pancreatic head and uncinate process approximating 4.3 cm x 3.7 cm. There was also irregular bowel wall thickening within the proximal transverse colon with nodularity extending into the lumen suspicious for tumor recurrence with concern for local invasion of adjacent mesentery with mesenteric lymph nodes noted. These findings and symptoms were consistent with possible pancreatic carcinoma versus retroperitoneal malignancy from the colon mass.

**Figure 1:**
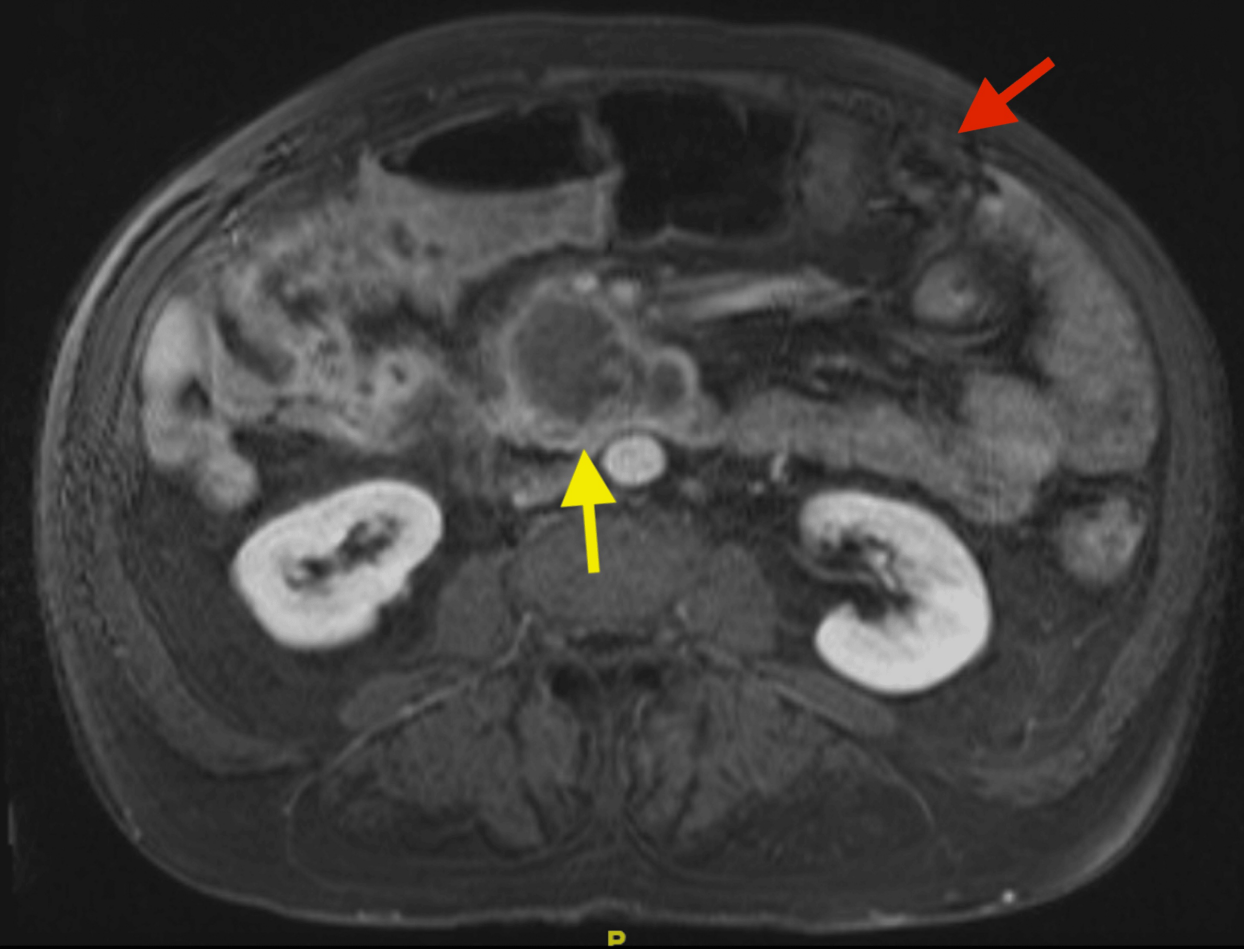
Magnetic Resonance Imaging of the Abdomen and Pelvis With Contrast Yellow arrow: Pancreatic head mass; Red arrow: Transverse colon mass at the prior partial colectomy anastomosis site.

Surgical oncology was consulted for evaluation of the possible colon cancer recurrence at the ileocolic anastomosis and concern for the retroperitoneal mass. Gastroenterology was consulted for an endoscopic evaluation with an EGD and colonoscopy. Repeat imaging was ordered prior to procedural evaluation. Magnetic resonance imaging (MRI) of the abdomen with contrast was significant for irregular colonic wall thickening at the anastomosis site, mesenteric soft tissue infiltration extending to the mesenteric root with a necrotic mass, a periduodenal/uncinate mass measuring 6.2 cm x 4.7 cm concerning for metastasis versus duodenal or pancreatic carcinoma, and an indeterminate hypervascular liver lesion in segment 5 measuring 6 mm (Figure 1).

Pertinent labs included a carcinoembryonic antigen (CEA) level of 3.0 ng/mL (reference: 0.0-2.5 ng/mL), cancer antigen 19-9 (CA19-9) level of 23 U/mL (reference: <34U/mL), and lipase 96 U/L (reference: 73-393 U/L). Liver function tests (LFTs) were within normal limits (Table 1).

**Table 1:** Pertinent Lab Values During Initial Encounter

Lab	Patient Value	Reference Range
Carcinoembryonic Antigen (CEA)	3.0 ng/mL	0.0-2.5 ng/mL
Cancer Antigen 19-9 (CA19-9)	23 U/mL	<34 U/mL
Lipase	96 U/L	73-393 U/L

**Figure 2:**
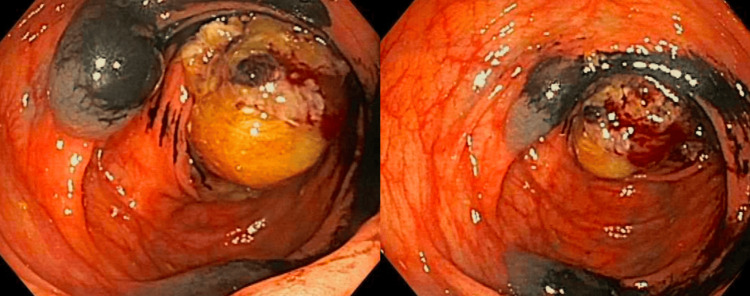
Diagnostic Colonoscopy Images Mass was observed during colonoscopy at the site of the previous partial colectomy anastomosis. Images were taken after the biopsy and tattooing of the area.

The patient underwent an EGD with EUS which revealed the left lobe of the liver had an area of abnormal echotexture. Due to the Roux-en-Y anatomy, visualization of the pancreatic head was limited and a biopsy was not able to be performed as the pathway to the biopsy site was too long for the endoscope. A colonoscopy was completed two days after the initial EGD due to poor bowel prep, and significant findings of a villous, fungating, infiltrative, sessile, ulcerated, and partially obstructing mass were identified on gross examination of the ileocolic anastomosis (Figure 2). The mass measured approximately 5 cm in length. Biopsies were obtained with a cold snare and the site was tattooed. The pathology of the colon mass returned as invasive moderately differentiated adenocarcinoma.

**Figure 3:**
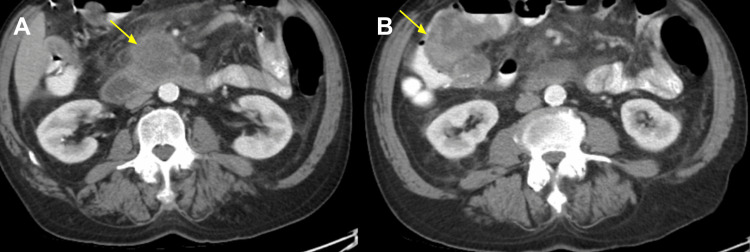
CT Abdomen Pelvis With and Without Contrast (A) Uncinate process mass abutting the duodenal C-loop with narrowing of the superior mesenteric vein (SMV), measuring 6.9 x 5.2 cm. (B) Right hemicolectomy anastomosis with a filling defect concerning tumor recurrence, measuring 7.4 x 6.6 cm.

A CT-guided biopsy of the pancreas was then performed after failure to collect sufficient samples with a traditional EGD and EUS. A CT of the abdomen and pelvis with and without contrast at the time of the biopsy demonstrated the hypodense mass in the root of the small bowel mesentery possibly arising from the uncinate process of the pancreas and measuring 6.9 x5.2 cm (Figure 3) with abutment and suspected infiltration of the duodenal C-loop. There was a high-grade narrowing of the superior mesenteric vein secondary to tumor burden as well as surrounding fat stranding.

The patient underwent a diagnostic laparoscopy with lysis of adhesions, omentectomy, partial colectomy/enterectomy, regional lymphadenectomy, ileocolic anastomosis, and cholecystectomy. The surgical pathology was significant for an 8.0 cm invasive moderately differentiated mucinous adenocarcinoma invading through the underlying muscularis propria to involve the pericolonic adipose tissue. The patient was then discharged with a plan for initiation of pembrolizumab and a positron emission tomography (PET) scan as an outpatient.

The patient returned two months later after the oncologist noted increased jaundice in the outpatient setting the day after the pembrolizumab infusion. The patient was admitted for inpatient evaluation. Labs on arrival during this second admission were significant for aspartate aminotransferase (AST) 69 U/L (reference: 15-37 U/L), alanine transaminase (ALT) 68 U/L (reference: 15-37 U/L), and total bilirubin 4.7 mg/dL (reference: 0.2-1.0 mg/dL) (Table 2). Due to previously failed biopsy attempts in the setting of the RYGB anatomy, the possibility of utilizing a modified EDGE procedure was discussed. The modification involves performing an EUS-FNA instead of the traditional ERCP typically included in the procedure.

**Table 2:** Pertinent Lab Values From Second Encounter

Lab	Patient Value	Reference Range
Aspartate Aminotransferase (AST)	69 U/L	15-37 U/L
Alanine Aminotransferase (ALT)	68 U/L	15-37 U/L
Total Bilirubin	4.7 mg/dL	0.2-1.0 mg/dL

**Figure 4:**
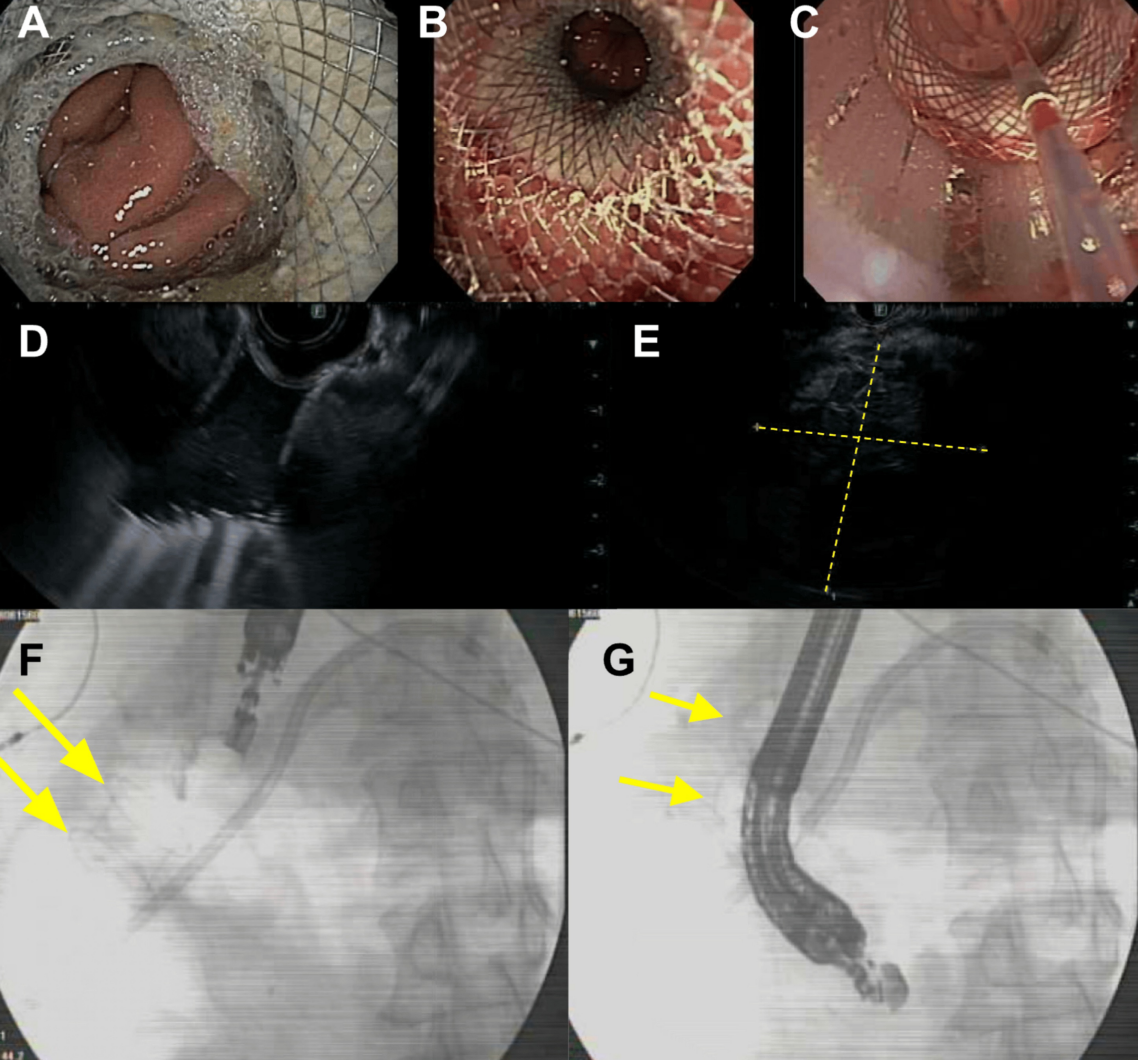
Endoscopic, EUS, and Fluoroscopic Images From EDGE Procedure (A & B) Stable gastrogastric tract with stent in place. (C) Fine-needle aspiration (FNA) needle being introduced through the gastrogastric tract for pancreatic mass biopsy. (D) Endoscopic ultrasound (EUS) of the gastrogastric stent tract. (E) EUS showing the pancreatic mass. (F & G) Fluoroscopy showing the endoscope approaching and traversing the gastrogastric tract through the lumen-apposing metal-covered stent. EDGE: Endoscopic ultrasound-directed transgastric endoscopic retrograde cholangiopancreatography

**Figure 5:**
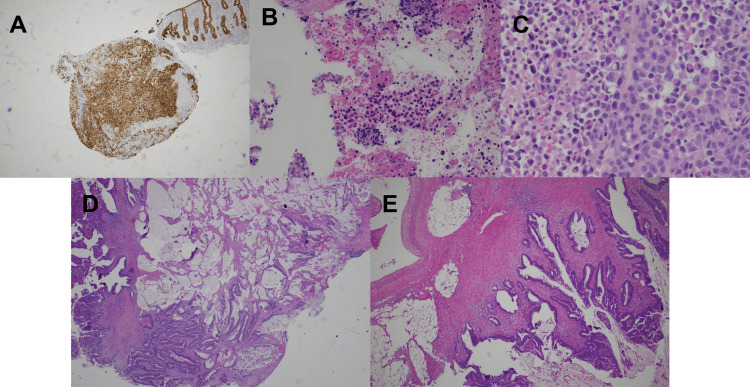
Histology of Pancreatic and Colon Mass (A) Cytokeratin staining of the pancreatic mass. (B) Hematoxylin and eosin (H&E) staining of the pancreatic mass. (C) Signet ring cells were observed on the H&E stain of the pancreatic mass. (D) Adenoma of the colon on H&E stain. (E) Mucinous adenoma of the colon.

A magnetic resonance cholangiopancreatography (MRCP) was performed and was significant for biliary obstruction and dilation due to the pancreatic head and an uncinate mass of 14 x 9 x 10 cm, a common bile duct (CBD) diameter of 2.3 cm, and proximal pancreatic duct of 6 mm. A CT-guided biopsy was attempted and unsuccessful. The patient then proceeded to have a staged internal EDGE procedure to obtain an endoscopic biopsy. The first stage of the EDGE was performed with a gastrogastrostomy created using a covered metal stent connecting the afferent and efferent portions of the stomach. Two days later, once the gastrogastric tract had time to mature, the EUS with transgastric FNA was performed to obtain a sample of the pancreatic mass (Figure 4). Samples were taken from the infiltrating duodenal mass and pancreatic head mass during the same procedure. Pathology results of the sample resulted in poorly differentiated adenocarcinoma with signet ring cell features (Figure 5).

Mismatch repair testing was completed on both samples testing for MLH1, PMS2, MSH2, and MSH6 in the colon mass with MSH6 being found to have a loss of expression, raising concern for HNPCC. The pancreatic mass was also tested for MLH1, PMS2, MSH2, MSH6, and gastric HER2 genomic changes but all were found to be present. The patient ultimately decided to stop pursuing aggressive treatment and was discharged to hospice for palliative management.

## Discussion

The benefits of using an EUS for the evaluation of pancreatic cancer are well-established in the literature with a sensitivity of 85-92% and specificity of 96-98% when using FNA [7]. This imaging and sampling method was called for in the setting of the multiple failed biopsy attempts prior to the modified internal EDGE as described in this case. The modification detailed in this case is the EUS-FNA that was completed after creating the gastrogastric tract with the LAMS, with the EUS-FNA replacing the traditional ERCP portion of an internal EDGE procedure. By creating a gastrogastric tract, the pancreatic mass was able to be successfully sampled using the capabilities of the EUS-FNA since the endoscope could now reach the previously isolated afferent limb of the stomach for a transgastric biopsy. This procedure allowed for the challenges of the elongated RYGB anatomic pathway to be bypassed by creating a shorter pathway that was well within the proximity of the endoscope allowing for adequate transgastric sampling of the mass.

The use of the internal EDGE to allow for the EUS-FNA biopsy to be accomplished is the aspect of this case that the authors believe to be a valuable contribution to the literature. The utility of the internal EDGE procedure for accessing difficult areas has been established, and EUS-FNA has also been proposed as superior to transcutaneous CT biopsy due to no risk of seeding the biopsy tract, which has been proposed as a possible complication with this method of sampling [10-12]. The modified EDGE described in this case still has the same risks associated with similar portions to a traditional EDGE, including intraprocedural LAMS migration/malposition (16%), bleeding (2%), persistent fistula (1%), and perforation (1%) per the systemic review done by Prakash et al. [13]. EUS-FNA also carries risks with it and the case detailed here would carry the same procedural risks including pancreatitis (0.92%), bleeding (0.69%), infection (0.44%), desaturation (0.23%), and perforation (0.21%) per the meta-analysis done by Zhu et al. [14]. Since RYGB patients have anatomic limitations to conventional endoscopic approaches, the ability to access the afferent limb endoscopically with the described modified EDGE procedure allowed for adequate testing and sampling of the otherwise inaccessible mass when previous sampling attempts failed. The primary limiting factor for this procedure is that the advanced endoscopic modalities require operator experience and should be conducted by those with experience in these procedures.

## Conclusions

The availability of literature describing a modified EDGE procedure with EUS-FNA being used to obtain viable samples for oncologic evaluation is limited. Thus, this case is a valuable addition to the literature as it demonstrates the utility of gastrogastric tract formation in allowing EUS-FNA in RYGB patients. Awareness of the utility of EDGE procedures and related techniques is crucial so that they can be considered when approaching the difficult anatomy of RYGB patients and also reduce potential seeding from transcutaneous biopsy methods. The authors believe additional research into the use of this modified internal EDGE procedure would help establish this method for use in attaining successful biopsies of pancreatic masses in patients with RYGB anatomy and thus influence current standards of practice.
